# Use of Virtual Reality in the Pediatric Perioperative Setting and for Induction of Anesthesia: Mixed Methods Pilot Feasibility Study

**DOI:** 10.2196/58905

**Published:** 2025-05-16

**Authors:** Yu Tong Huang, Sofia Addab, Gianluca Bertolizio, Reggie Hamdy, Kelly Thorstad, Argerie Tsimicalis

**Affiliations:** 1 Faculty of Medicine and Health Sciences, McGill University, Montreal, QC, Canada; 2 Clinical Research, Shriners Hospitals for Children–Canada, 1003 Decarie Blvd, Montreal, QC, H4A 0A9, Canada, 1 5148424464; 3 Division of Pediatric Anesthesia, The Montreal Children's Hospital, McGill University Health Center, Montreal, QC, Canada; 4 Division of Pediatric Surgery, The Montreal Children's Hospital, McGill University Health Center, Montreal, QC, Canada; 5 Faculty of Dental Medicine and Oral Health Sciences, McGill University, Montreal, Canada

**Keywords:** virtual reality, augmented reality, mixed reality, extended reality, computer-generated simulation, digital world, virtual environment, anxious, pediatrics, anesthetics, preoperative, feasibility, artificial intelligence, digital health technology, surgery, child care

## Abstract

**Background:**

Children commonly experience high levels of anxiety prior to surgery. This distress is associated with postoperative maladaptive behaviors. Virtual reality (VR) is an innovative tool for reducing anxiety and pain during various medical procedures. Previous randomized controlled trials have demonstrated its efficacy in reducing children’s anxiety in the preoperative waiting room or during induction.

**Objective:**

The primary aim of this study was to examine the feasibility of VR distraction throughout the perioperative period, from the waiting room until the induction of general anesthesia (GA). Secondary aims were to assess its clinical utility, tolerability, and initial clinical efficacy.

**Methods:**

A mixed methods, concurrent triangulation feasibility trial was piloted at the Shriners Hospitals for Children–Canada. Participants played an interactive VR game throughout the perioperative period, starting from the waiting room until induction. Feasibility was examined with the duration of the VR intervention, recording the number of interruptions, and taking field notes. Clinical utility was assessed using a perception questionnaire. Tolerability was evaluated by the Child Simulator Sickness Questionnaire (CSSQ). Initial clinical efficacy was assessed by the Faces Pain Scale–Revised, Faces Anxiety Scale, Graphic Rating Scale for multidimensional pain, the Induction Compliance Checklist, and the Pediatric Anesthesia Emergence Delirium scale. Quantitative data were supported with field notes and semistructured interviews with patients and parents. Quantitative and qualitative themes were compared via the triangulation protocol to produce final themes.

**Results:**

A total of 39 patients, with a mean age of 11.9 (SD 2.8) years, undergoing elective surgery under GA participated in the study. Stakeholders, including patients, parents, and health care providers, were receptive and willing to adapt to VR. Of the 39 patients, 19 (49%) continued to use VR during transportation and 6 (15%) were induced with VR. Barriers to feasibility included (1) interruptions to VR in 92% (36/39) of patients by health care professionals, (2) unpredictable surgery delays prolonging the duration of the VR intervention (mean 23.1, SD 24.4 minutes; range 5‐150 minutes), and (3) discontinuation of VR before induction due to mask seal (n=3) and discomfort with supine positioning (n=2). Patients were generally satisfied with VR, deemed it acceptable and easy to use, and would recommend it to others. VR was tolerable with no self-reported simulator sickness (CSSQ: mean 0.01, SD 0.1). The mean Faces Anxiety Score was 1.5 (SD 1.1) at baseline and 0.7 (SD 0.9) during VR.

**Conclusions:**

While VR demonstrated good clinical utility and was well tolerated in the broad perioperative setting, this study highlighted important feasibility barriers in the waiting room and especially during induction of anesthesia, both at the organizational and technical levels. This study highlights several considerations that should be carefully addressed for the successful implementation of perioperative VR.

## Introduction

Virtual reality (VR) is an innovative tool for managing anxiety and pain during medical procedures, such as needle insertion, dressing changes, and dental care [1]. Up to 60% of children experience high levels of anxiety at induction of general anesthesia (GA) [2,3]. This distress is associated with a greater risk of postoperative emergence delirium [4,5], disturbed sleep, and behavioral and emotional disturbances [6]. Previous studies and a meta-analysis have demonstrated the efficacy of preoperative operating room (OR) tours by VR in reducing anxiety [7-18]. With regard to VR use during induction of anesthesia, 2 randomized controlled trials (RCTs) by Jung et al [19] and Samnakay et al [20] have demonstrated the efficacy of a VR game for distraction and anxiety reduction compared to standard care or noninferiority to the use of a 2D tablet. The perioperative period is a continuum of multiple moments that can cause anxiety, including awaiting surgery in the waiting room, being transported to the OR, and undergoing induction of anesthesia in the OR. Understanding the VR feasibility for distraction across these different moments would be fundamental to determining if and where VR can be integrated into the overall perioperative patient flow. Integrating VR into the induction of anesthesia may be more technically complex than in other studied settings, such as intravenous (IV) insertions. Hence, the primary objective of this study was to determine the feasibility of using VR for distraction in the perioperative setting, from the preoperative waiting room to induction. Secondary aims were to assess the clinical utility, tolerability, and initial effectiveness of VR in the same time frame.

## Methods

### Study Design and Setting

A mixed method [21], concurrent triangulation feasibility study was piloted on the OR floor of the Shriners Hospitals for Children–Canada, a university-affiliated, not-for-profit, bilingual, pediatric orthopedic hospital located in Montreal, Quebec, Canada. The OR floor consisted of a preoperative waiting room, 4 ORs, and a postanesthesia care unit (PACU). In this study, 14 anesthesiologists, 17 surgeons, 14 nurses, 3 respiratory therapists, and 4 orderlies were involved in VR use. The study commenced during the COVID-19 pandemic, resulting in parents no longer being allowed in the OR during the anesthesia induction, with recruitment starting in May 2021 and ending in June 2022.

### VR-CORE Outcomes for VR2 Trials

The VR Clinical Outcomes Research Experts (VR-CORE) methodological framework guided this VR2 trial, of which the aim is to produce an “initial assessment in the target patient population within a representative clinical setting” [22]. The primary outcome was the feasibility of perioperative VR, consisting of barriers and facilitators to this intervention [22]. Secondary outcomes consisted of: (1) clinical utility, defined as acceptability, ease of use and understanding, satisfaction, and recommendation of the VR intervention; (2) tolerability, which entailed the absence of physical or emotional adverse effects related to VR; and (3) initial clinical efficacy, defined as patients’ outcomes of anxiety, pain, and compliance at induction.

### Participants

Convenience sampling was used to prospectively recruit participants in the preoperative evaluation clinic. Patients were eligible if they (1) were aged between 5 and 21 years, (2) had a scheduled elective surgery under GA, and (3) could understand French or English. Patients were excluded if they (1) had a cognitive, auditory, or visual impairment preventing VR use or (2) had a history of seizures or epilepsy. Parents or legal guardians were eligible if they were present with the child and were willing to share their perspectives. A sample size of approximately 40 patients was based on a prior feasibility study including at the study site [23] and a similar setting [24]. The sample size aligned with the VR-CORE recommendations [22].

### VR Intervention

Participants played a pretested [23,25,26], interactive game with sound, DREAM (Paperplane Therapeutics, Inc) [27], via the Pico Neo 3 headset. DREAM entails patients throwing red balls at balloons, diamonds, and trolls in a fantastical landscape. The game was developed with input from medical professionals and tested at pediatric sites, including the study site [23,25,26]. DREAM was designed for health care use, allowing for (1) reduced speed to prevent VR sickness, (2) one hand for play, (3) head movement to orient the character, (4) aesthetic appeal, and (5) a no “loss” state. One headset was available for this study. The game was not mirrored onto a tablet for parents or clinicians to view to avoid internet connection-related interruptions to gameplay.

### Study Procedure

**Figure 1.**
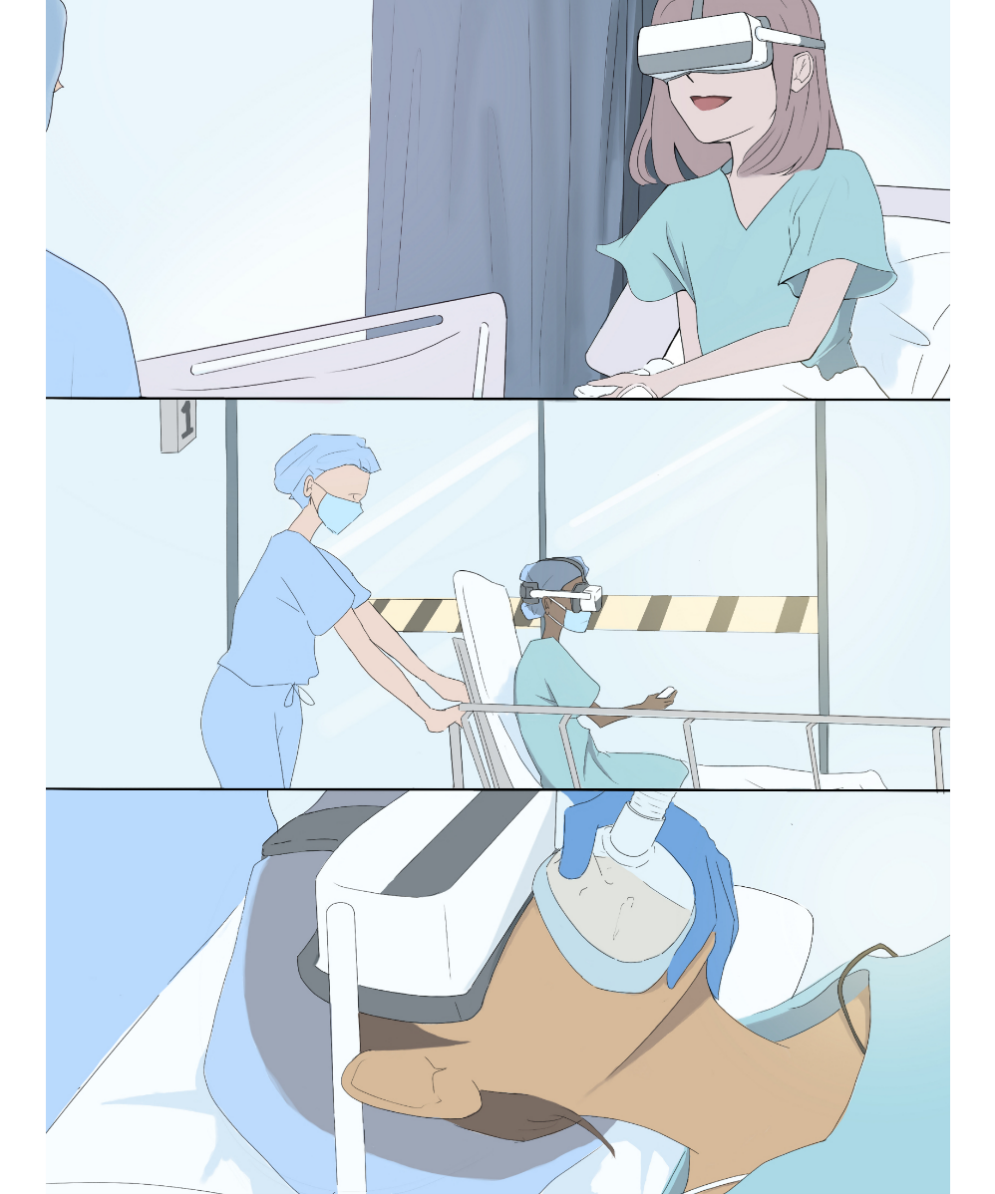
Virtual reality intervention at various perioperative time points: in the waiting room (top), during transport (middle), and during induction (bottom).

Nursing staff in the preoperative clinic helped identify eligible participants. A member of the research team explained the study to parents and patients and, if agreeable, obtained informed consent and assent during their preoperative appointment, days or weeks before their surgery. On the day of surgery, game instructions were provided, and the headset was fitted to the patient by a researcher in the preoperative waiting room. The VR intervention was offered within the workflow of the study site (Figure 1). At least 5 minutes of gameplay for VR immersion was encouraged before transfer to the OR; however, gameplay was allowed to extend for longer durations if there were OR delays. Patients were encouraged to pause after 30 minutes of screen time to avoid VR sickness. The researcher remained on standby and troubleshooted VR issues. Patients could pause or discontinue VR at any point. Nurses, orderlies, surgeons, and anesthesiologists usually visited patients in the waiting room to perform preoperative tasks and assessments before transfer to the OR. At the study site, patients routinely received Tylenol and did not routinely receive anxiolytics unless deemed necessary. EMLA cream was routinely applied 30 minutes prior to awake IV insertions. Patients were verbally notified when it was time for their surgery and were asked if they wanted to continue VR for transfer to the OR and induction. Patients were preoxygenated with an age-appropriate mask. The method of induction, usually inhalational with sevoflurane at the study site, was left to the anesthesiologist’s discretion. After induction, the headset was removed.

### Data Collection

After informed consent, baseline sample characteristics were collected from patients and hospital charts. Patient-reported anxiety, pain, and VR sickness were subsequently collected in the waiting room using the Faces Anxiety Scale (FAS) [28], the Faces Pain Scale–Revised (FPS-R) [29], and the Child Simulator Sickness Questionnaire (CSSQ) [30], respectively. During the VR intervention, field notes were taken by a researcher [23]. In the OR, induction compliance was assessed by a researcher via the Induction Compliance Checklist (ICC) [31]. In the PACU, as part of standard practice, the nurses recorded the emergence delirium using the Pediatric Anesthesia Emergence Delirium (PAED) scale [4]; the scores were retrieved from the chart. Following the surgery, patients retrospectively reported anxiety, pain, and VR sickness experienced during VR use using the FAS, FPS-R, and CSSQ, as well as the Graphic Rating Scale (GRS) [32]. Patient perception was assessed using a modified version of the Patient Perception Questionnaire [33]. These data were collected either immediately after surgery in the PACU, a few days later in the inpatient unit, or weeks to months later at the follow-up appointment. Finally, if agreeable, the patient and their parent (if present) participated in an audio-recorded, semistructured interview, using a previously used interview guide created by the study team [23].

### Data Analysis

Descriptive statistics were used to summarize the sociodemographic and instrument data using Microsoft Excel (2016) measures of central tendency and variance, generating a list of key findings set aside for triangulation. Qualitative data analysis was conducted separately through directed content analysis [34] of the field notes and interviews. The themes identified were supported by quotes, observations, and field notes. Triangulation analysis of qualitative and quantitative data led to the identification of meta-themes, which resulted from qualitative and quantitative sources, and themes drawn from one data source [23,35]. Through this process, qualitative and quantitative data were compared and contrasted, resulting in “agreement,” “disagreement,” “silence,” or “complementarity” between the two data source.

### Ethical Considerations

This study was performed in accordance with the principles of the Declaration of Helsinki. Approval was granted by the McGill Institutional Review Board (A06-M31-19B). Informed consent was obtained from all participants and legal guardians included in the study. All data were deidentified prior to analysis to maintain participant privacy. Participants received no monetary compensation.

## Results

### Sample Characteristics

**Figure 2.**
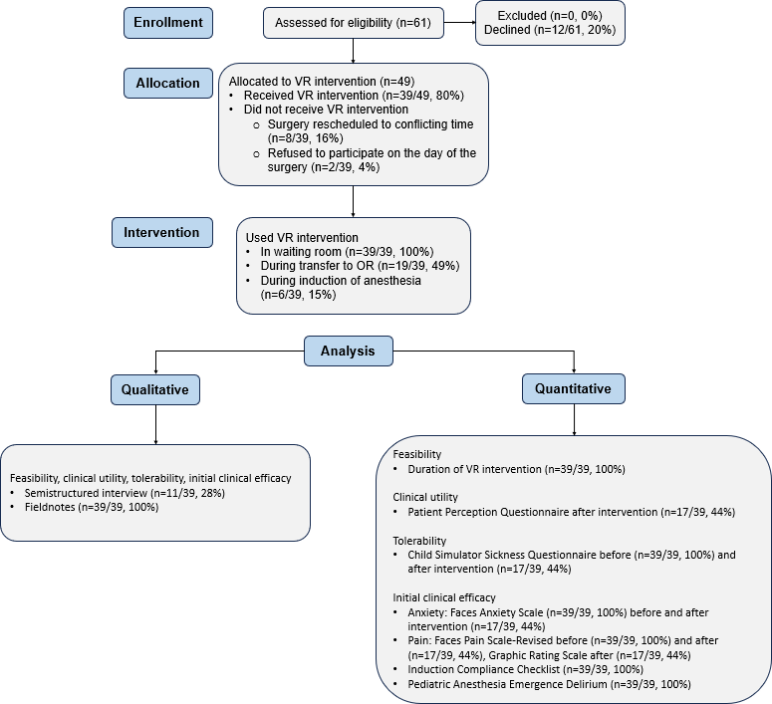
CONSORT (Consolidated Standards of Reporting Trials) flow diagram. OR: operating room; VR: virtual reality.

In total, 61 eligible patients were approached for study participation, of which 49 consented or assented to participate. Two patients withdrew on the morning of their scheduled surgery before the VR intervention, as they were no longer interested in using VR. Eight patients were lost to follow-up as their surgery was canceled, and their rescheduled date conflicted with other participants (Figure 2). Overall, 39 patients, with a mean age of 11.9 (SD 2.8) years and a median age of 12 (IQR 10-13.5) years, used VR in the perioperative setting, for a participation rate of 64% (39/61; Table 1). A total of 11 participants and 9 parents agreed to participate in the interview following their surgery. The remaining patients were lost to follow-up after their surgery.

**Table 1.** Patient demographics (n=39).

Characteristics	Values
Age (years)	
Mean (SD)	11.9 (2.8)
Median (IQR)	12 (10‐13.5)
Sex, n (%)	
Male	18 (46)
Female	21 (54)
Race, n (%)	
White	30 (77)
Black	5 (13)
Hispanic	1 (3)
Other	3 (8)
Patients receiving preoperative medications, n (%)	
Tylenol	39 (100)
Midazolam	2 (5)
Diagnosis, n (%)	
Hip and leg disorders	10 (26)
Sports injuries	7 (18)
Foot and ankle disorders	5 (13)
Scoliosis	4 (10)
Abdominal	3 (8)
Bone and soft tissue tumors	2 (5)
Neuromuscular	2 (5)
Other	6 (15)
Surgery, n (%)	
Orthopedic	
Hip and knee	15 (38)
Hardware removal or ablation	8 (21)
Foot and ankle	5 (13)
Spine	4 (10)
Other	4 (10)
General surgery	
Hernia repair	2 (5)
Urology	
Excision of penile cyst	1 (3)

### Feasibility

#### Overview

**Figure 3.**
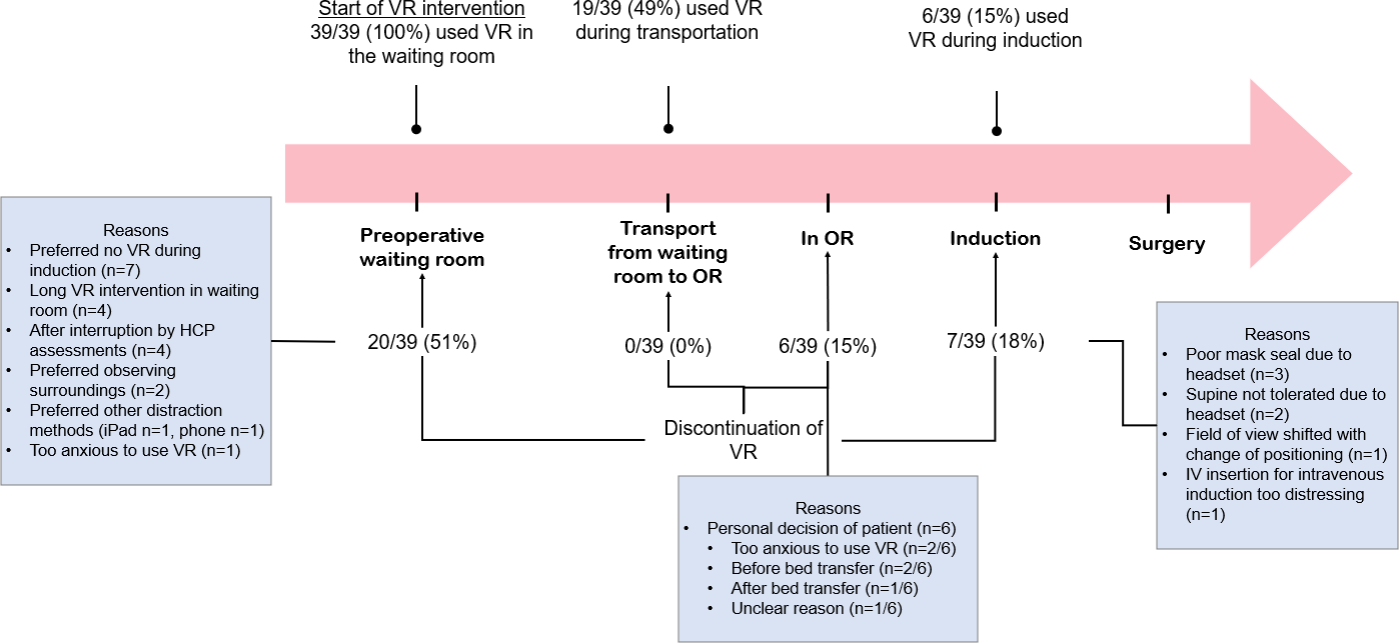
Timeline of VR intervention and reasons for discontinuation of VR. HCP: health care professional; OR: operating room; VR: virtual reality.

Of the 39 patients, 6 (15%) used VR across the entire perioperative period, with 20 (51%) discontinuing VR in the preoperative waiting room, 6 (15%) inside the OR before induction, and 7 (18%) during induction (Figure 3). Transportation to the OR proceeded smoothly with no discontinuations. Most inductions were inhalational (37/39, 95%). Two (5%) IV inductions were attempted with VR, one of which was discontinued due to anxiety.

#### Facilitator: Receptiveness and Adaptability

Health care professionals appeared enthusiastic about the VR intervention, encouraging the patients during gameplay and offering implementation suggestions to the research team. On 6 occasions, the clinician adapted their preoperative evaluation in the waiting room to minimize interruption to the VR intervention by speaking to the parents or by discretely performing the task while the patient continued playing. After explaining the VR intervention and its implications for induction, all anesthesiologists attempted to integrate VR into their workflow. For example, the head of the OR table was raised, or extra pillows were placed under a patient’s head for comfort while wearing the headset during induction. Only 2 (14%) of the 14 anesthesiologists in this study had prior VR experience.

#### Facilitator: Communication

Communication among patients, parents, and clinicians was maintained during the VR intervention. Clinicians explained what they were doing and notified the patient before important time points, such as leaving the waiting room for the OR. Clinicians and parents were actively involved in the VR intervention, asking children what they were seeing. Their involvement fostered a positive environment for patients to be immersed in VR. Patients could easily notify their clinicians about discomfort or desire to pause VR. Patients generally appreciated being informed of what was happening when using VR. One patient who was induced with VR reflected, “I would have liked to be notified when they were going to put on the induction mask. They didn’t tell me! And I was surprised!” [Participant 29].

#### Barrier: Interruptions to the VR Intervention

Most patients (36/39, 92%) experienced at least one interruption during their entire VR intervention (from waiting room to induction), causing most patients (34/39, 87%) to remove their headset at least once. In the preoperative waiting room, the most common reason for interruption was preoperative assessments by clinicians (34/39, 87%), after which few patients (4/39, 10%) discontinued VR altogether. One mother stated, “[My daughter] was saying that she was so relaxed, and that VR helped her think about other things. And you could see that she was immersed in the game. And then, at one point, the effect was kind of lost because doctors came to see her” [Mother of participant 32].

#### Barrier: Duration of VR Use

The average total duration of the VR intervention was 23.1 (SD 24.4) minutes, ranging from 5 to 150 minutes. Most patients (37/39, 95%) had sufficient VR playtime in the preoperative waiting room to achieve immersive distraction for induction. However, due to frequent, unpredictable delays in the OR schedule, playtime in the waiting room was often extended for longer durations. Hence, some patients (15/39, 38%) took breaks in the middle of VR, and a few patients (4/39, 10%) became tired or bored, discontinuing VR altogether in the waiting room (Figure 3). One parent shared, “[…] at one point he stopped playing because it was always the same thing. After a while, it was enough” (parent of participant 10). In contrast, on one occasion, a patient [Participant 16] arrived late, leading clinicians to prioritize preoperative evaluations and reducing VR playtime to only 2 minutes before transfer to the OR. Nevertheless, this patient did not exhibit anxiety behaviors and had excellent compliance during induction (ICC=0).

#### Barrier: Induction With VR

Some challenges were noted in integrating VR into the intraoperative workflow. Among the 13 patients who attempted induction with VR, 6 (46%) inductions, including one IV induction, were completed with VR (Figure 3). VR was discontinued during 7 attempted VR inductions (7/13, 54%), including one IV induction, revealing challenges such as poor mask seal with the headset (n=3) and discomfort with supine positioning due to the headset structure (n=2; Figure 3). Achieving a good mask seal and ensuring a quick induction were prioritized by anesthesiologists over VR use, sometimes leading to VR discontinuation. Additionally, even when the patient kept the headset during inhalational induction, one anesthesiologist explained that it was somewhat difficult to maintain a good mask seal. Additionally, 3 patients discontinued VR during the transfer from the stretcher to the OR table, and 2 temporarily paused VR. One patient explained that it felt like they were falling during the transfer from the stretcher to the OR bed with the headset.

#### Barrier: Technical Issues

VR-related technical issues, namely loss of audio (5/39, 13%), headset adjustments (5/39, 13%), and changes in the field of view when the patient changed orientation (4/39, 10%), were other sources of interruptions, at which point the researcher was able to quickly troubleshoot the issue, allowing for the patient to resume playing. On one occasion, the headset ran out of battery in the OR due to extended preoperative play time, causing a delay while the charger was retrieved, after which the patient continued to play.

### Clinical Utility

#### Acceptability

VR was accepted by patients and parents. Almost all patients (38/39, 97%) were initially willing to use VR. Many patients looked forward to playing VR, asking the front desk personnel and nurses to commence upon arrival at the hospital. Parents supported the integration of VR in their child’s care, asking them about the game and inquiring about hospital implementation efforts.

#### Satisfaction

Overall, all patients found VR fun (Table 2) and were happy to use VR perioperatively for distraction (Table 3), particularly in the preoperative waiting room. Several parents remarked their child was “gone in another world.” One patient said, “I hope this never ends. Oh my god, this is so much fun” [Participant 20]. In contrast, adolescents primarily discontinued VR in the waiting room despite acknowledging the distraction it provided due to the game’s repetitive and puerile nature. A few patients (3/39, 8%) opted to use other nonpharmacological means of distraction, such as their phone.

**Table 2.** Mean Graphic Rating Scale score for each item.

	Score, mean (SD)^a^
Pain	
Time spent thinking about pain	0 (0)
Unpleasant pain	0.1 (0.5)
Worst pain	0.4 (1.5)
Fun	7.8 (1.6)
Nausea	0 (0)

a Each item is rated on a 10 cm line, from 0 to 10. Along the line, descriptive markers “mild,” “moderate” and “severe” are present.

**Table 3.** Patient perceptions of the clinical utility of the virtual reality intervention (n=17).

Scale and items	Responses, n (%)				Quotes
	1	2	3	4	
1=Not at all, 2=A little bit, 3=Some, and 4=A lot					
How much did the virtual reality game distract you during your medical procedure?	1 (5.9)	2 (11.8)	6 (35.3)	8 (47.1)	“When they put me to sleep, I didn’t even feel like I was being put to sleep. All I remember is having the mask on my face, being told to breathe, and then I was gone” [Participant 27].
How much did the virtual reality game help lower your pain during your medical procedure?	11 (64.7)	1 (5.9)	2 (11.8)	3 (17.6)	“I didn’t have pain to begin with.” [Participant 32] “Since I was focused on [VR], I would say that my pain [behind my knees] decreased.” [Participant 1]
1=Very unlikely, 2=Unlikely, 3=Likely, and 4=Very likely					
Would you ask to play a virtual reality game for your next medical procedure?	0 (0)	0 (0)	9 (52.9)	8 (47.1)	N/A
Would you recommend playing a virtual reality game to another patient like you?	0 (0)	0 (0)	5 (29.4)	12 (70.6)	“This game would be good even for people my age who don’t know much about video games. […] There are some more anxious, or who are having trouble coping, or for who it’s their first surgery. VR would help them.” [Participant 1]
1=Very unhappy, 2=Unhappy, 3=Happy, and 4=Very happy					
How happy were you with playing the virtual reality game during your medical procedure?	0 (0)	0 (0)	10 (58.8)	7 (41.2)	“I hope this never ends! Can I have this for my birthday?” [Participant 20] “Mom, I won 2585 points!” [Participant 44] “It was okay for my age. It’s not the best thing ever, but […] to distract me, it’s pretty good.” [Participant 27]

#### Ease of Use and Understanding

All patients, regardless of age and previous experience with VR or video games, rapidly understood how to play the game easily. Older patients were pre-emptively instructed by the researcher on troubleshooting certain technical issues, such as shifts in the field of view during position changes.

#### Recommendation of the VR Intervention

All patients would request and recommend VR if they or another patient needed another surgery under anesthesia (Table 3). One patient aged 17 years explained, “This game would be good even for people my age who don’t know much about video games. […] There are some more anxious, or who are having trouble coping, or for whom it’s their first surgery. VR would help them” [Participant 1].

### Tolerability

#### Physical Adverse Events

All patients who used VR, regardless of duration, experienced no VR sickness at baseline, or during VR, as per the CSSQ (Table 4) and the GRS (Table 2). Two patients felt that their eyes were tired and took a break. One patient felt “a little bit dizzy,” prompting him to take multiple short breaks in VR use in the waiting room. For the majority of children, the VR headset was comfortable. One patient found the headset “a little bit heavy on [her] head,” which resolved when it was loosened. For another, the headset sometimes slid down his face. Discomfort was felt at the back of the head with 4 patients when asked to lay supine for induction with VR. One patient described a sensation of falling when being transferred from one bed to another with VR.

**Table 4.** Anxiety, pain, and virtual reality (VR) sickness: baseline versus during VR intervention.

Scale	Score, mean (SD)	
	Baseline (n=39)	During VR (n=17)
Faces Anxiety Scale^a^	1.5 (1.1)	0.7 (0.9)
Faces Pain Scale–Revised^b^	0.3 (0.9)	0.3 (1.2)
Child Simulator Sickness Questionnaire^c^		
Nausea	0 (0)	0 (0)
Oculomotor	0 (0)	0.02 (0.14)
Disorientation	0 (0)	0.02 (0.14)
Total	0 (0)	0.01 (0.13)

a The Faces Anxiety Scale for children is scored from 0 to 4, showing 5 faces with increasing levels of anxiety. A score of 0 means “no anxiety,” a score of 4 means “extreme anxiety.”

b The Faces Pain Scale–Revised is scored from 0 to 10, showing 6 faces with increasing pain. A score of 0 means “no pain,” a score of 10 means “very much pain.”

c A score of ≥3 of the Child Simulator Sickness Questionnaire indicates the presence of simulator sickness.

#### Emotional Adverse Events

The use of VR generated minimal adverse emotions. One patient [Participant 36], initially reluctant to use the headset due to a desire to see his surroundings, became immersed and distracted with reassurance.

### Initial Clinical Efficacy

#### Anxiety

At baseline, the mean FAS score was 1.5 (SD 1.1), and many patients (24/39, 62%) demonstrated anxiety-related behaviors such as restlessness, crying, maintaining proximity to parents, and tense body language. Of the 39 patients, 2 (5%) were premedicated with midazolam prior to using VR due to particularly elevated anxiety (Table 1). During the VR intervention, the FAS score was 0.7 (SD 0.9) (Table 4), and some patients visibly relaxed as they became immersed, laughing and making exclamations about the game: “Wow! There’s lots of big balloons!” Patients expressed VR helped them cope: “It was fun, it made me stop thinking about the surgery completely” [Participant 27]. Parents echoed the sentiment, saying “[VR] definitely worked,” [Mother of participant 15]. Others viewed VR more pragmatically, describing VR as a tool that “allows [them] to pass time” [Participant 1] rather than management of anxiety. Patients who used VR during induction overall agreed that it distracted them, “I don’t remember what [health care professionals] were doing [during induction]” [Participant 25], “When they put me to sleep, I didn’t even feel like I was being put to sleep. All I remember is having the mask on my face, being told to breathe, and then I was gone” [Participant 27].

Spikes of anxiety, displayed as crying, verbal expressions of fear, tense body language, and withdrawing, were observed before transport to the OR and before initiating induction. Signs of poor immersion included decreased head movement and letting go of the controller. Due to increased anxiety, 1 patient discontinued VR in the waiting room before leaving for the OR, and 2 patients discontinued VR in the OR.

#### Induction Compliance

The majority of patients (32/39, 82%) had a perfect anesthetic induction (ICC=0), 5/39 (13%) patients had a suboptimal induction (ICC=1 to 4), whereas 2/39 (5%) patients had a poor induction (ICC>4). One patient [Participant 37] with a poor induction (ICC=9) was immersed in VR in the preoperative waiting room but, upon removing his headset in the OR, became rapidly anxious and agitated during induction.

#### Emergence Delirium

Upon recovery in the PACU, there was no report of emergency delirium. All patients scored zero on the PAED scale.

#### Pain

The majority of patients (25/39, 64%) had low baseline pain scores (Table 4) and did not perceive VR to help with pain management at all (Table 3). In this study, patients were not subject to painful interventions except for a preinduction IV insertion in two cases, one of which was aborted due to anxiety (Figure 3). In contrast, patients with baseline pain associated with their condition agreed that VR helped decrease it, “Because I was concentrated on other things, the pain decreased” [Participant 1].

## Discussion

### Principal Findings

Overall, while VR showed good clinical utility and tolerability, our study demonstrated feasibility challenges with the implementation of VR in the waiting room and induction. Importantly, there was a 50% discontinuation rate prior to arrival at the OR. In the waiting room, notable challenges included interruptions to VR by health care professionals in almost all patients and OR scheduling delays leading to unexpectedly long durations of VR use in the waiting room. This issue was compounded by the fact that DREAM was designed for short procedural distraction, limiting its suitability for longer durations of use when ORs were delayed. In contrast to other studies, our study contained patients in the adolescent age group, up to 18 years old, who compared to younger children may find VR less “novel” and have preferences for more complex games than DREAM. Additionally, the discontinuation rate was likely influenced by the study design and philosophy of care, which advocates for patients deciding if and how they would like to use VR, and to encourage the integration of their other coping strategies to relieve their anxiety, which is reflective of the real-world use of VR in practice. In other studies using VR at induction, premature discontinuation rates for VR were much lower, around 10% or less [19,20,23,24,26,36]. However, these studies introduced VR right before transport to the OR, whereas in this study, VR was often started in the waiting room, more than 5 minutes before transport to the OR, whether intentionally at the request of the patient or unintentionally due to delays.

Despite these feasibility barriers in the waiting room, VR demonstrated good clinical utility, as patients and parents reported high satisfaction and enjoyment with the VR intervention. They all recommended VR for others to use and desired to use VR again in the health care setting. FAS was relatively low at baseline in our study population and did not appear to change “during intervention”, though statistical significance and causality were not assessed for in this feasibility study. Previous VR studies in the waiting room have yielded positive results for anxiety reduction [7-16]. However, since these studies did preoperative OR tours by VR, establishing comparisons with our study and game may be difficult. Overall, waiting room VR games can be a valuable tool for temporary immersion and distraction; however, the suitability of VR becomes limited in cases of prolonged wait times.

The interpretation of the feasibility and use of VR during induction is limited by the high discontinuation rate in the waiting room and the consequently small sample of participants (6/39, 15%) induced with VR. Nevertheless, we noted that while VR was beneficial for some patients, for others, the distraction afforded by VR became limited as their anxiety increased in the OR and during induction. Thirteen inductions were attempted with VR in our study, half of which were completed without interruption of VR. Difficulties with mask fit and supine positioning were major feasibility barriers. The Pico Neo 3 headset used in this study has a hard piece of plastic at the back of the head, which rendered supine positioning uncomfortable for some patients. Troubleshooting included additional pillows for padding and raising the head of the bed. As for troubles with the mask seal, the headset had to be propped up to allow access to the nose and mouth. In another study, anesthesiologists rotated the mask 180 degrees, allowing for a better fit with the headset at the expense of the mask seal [20].

To our knowledge, 2 RCTs have assessed the efficacy of VR during induction of anesthesia. Samnakay et al compared a VR video to a 2D video tablet, demonstrating similar efficacy between both technologies in reducing anxiety during induction. While children had higher satisfaction ratings with VR than with 2D tablets, anesthesiologists favored the 2D tablet over VR for inhalational inductions [20]. This is somewhat consistent with our findings, as we found induction with VR to be a technically challenging task that requires further optimization in technique and hardware, while satisfaction ratings remained high among patients. The similar efficacy of tablets and VR in their study [20], combined with the relative complexity of VR use during inhalational induction, argues against the use of VR during induction, though further evidence is needed to support this conclusion. Jung et al [19] demonstrated that a VR game, similar in mechanics to DREAM, during induction significantly decreased anxiety compared to the standard of care. In their study, only 1 out of 81 discontinued VR due to battery depletion, and 2 out of 81 discontinued VR during induction to see their parents [19]. This success, in contrast to our study, may be partly attributable to the use of a different headset (ie, Samsung Gear VR), in which the head strap is made of a softer, thinner material, not hindering supine positioning, and potentially to the health care professionals’ experience levels with using VR.

Most (32/39, 82%) patients displayed perfect induction compliance as per the ICC, the interpretation of which becomes limited by the low number of patients wearing the VR headset at induction (6/39, 15%). Of note, one patient displayed poor induction compliance (ICC=9) only once the headset was removed in the OR. This is probably explained by their known prior history of high anxiety and poor induction compliance in the perioperative setting and the limited benefits that VR may offer certain patients. Furthermore, we observed that conflicting stimuli from the “real” environment, such as transfers from stretcher to OR bed and exposure to volatile anesthetics, can remove patients from their immersion. Interestingly, Samnakay et al reported children with VR had lower odds of having a perfect induction compared to children with tablets. Because VR hides the real-world environment, it is possible that real-world stimuli generate unintended surprises [20]. OR tours by VR in the waiting room improved induction compliance in two studies at the same institution [9,10], whereas they did not in two other studies [7,19]. In this study, a subset of patients preferred observing the OR environment during induction. This brings into consideration a potential advantage of augmented reality (AR) for them, in which a digital image is overlaid in the real world. The use of AR may significantly reduce anxiety in pediatric patients [37] and improve mask acceptance in children undergoing induction of GA compared to children induced without [38].

### Clinical Implications

VR offers an innovative approach to help patients manage their anxiety before surgery under GA, but it is not a one-size-fits-all solution, and patients should be thoughtfully selected, especially considering the technical challenges encountered during induction. Importantly, the institution must be well organized for a coordinated approach to VR implementation. For minimal workflow and VR interruption, the intervention should ideally be started after completing all preoperative assessments. Ongoing collaboration and cooperation with all the health care providers should be elicited to minimize interruptions during gameplay. Indeed, a policy and procedure should detail when to alert cases of potential VR use with the health care team, especially anesthesiologists and respiratory therapists, such that they may adapt their approach and determine if VR is medically contraindicated. Prior to starting VR, the health care team should clarify with the patient when they would like to use VR, establish a communication plan, and determine whether the patient prefers being immersed or aware of their surroundings. However, they may always change their mind. It would be crucial to determine how VR for induction can be coordinated with expected and unexpected surgery delays. Depending on context, one health care professional should be responsible for administering and monitoring the VR intervention from the waiting room to the OR. Child life specialists, anesthesiologists, or respiratory therapists may be best equipped with that task as they are closely involved with the patient before and during induction. To render VR more compatible with induction, the health care team should opt for a headset that is not bulky, does not cover the patient’s nose or mouth, and has no counterbalance weight at the back. Anesthesiologists should be aware that mask fit with the headset may be suboptimal, and that access to eyes is limited. The feasibility of VR may improve as the institution and its clinicians become increasingly experienced with its use.

### Limitations

Due to the high discontinuation rate of VR preinduction, either by choice, surgical delays, or other circumstances, more data are needed to elucidate the feasibility of VR during anesthesia induction. Furthermore, many patients were lost to follow-up after their surgery; hence, self-reported postoperative outcomes such as anxiety and pain were incomplete. FAS “during VR” were obtained postintervention, in the PACU at the earliest, relying on the recall of the patients. Further, interviews were not conducted with all patients, potentially missing important insights for discontinuing VR use before induction. As this was a pilot feasibility VR2 trial, only descriptive statistics were performed, establishing no causal links. While this study included the perspectives of patients and parents, clinicians’ perspectives were obtained via field notes, limiting our ability to offer a complete picture of VR benefits and limitations. The VR game DREAM was designed for young children during acute medical procedures, with some of the older patients expressing boredom leading to discontinuation. Finally, the significant portion of the playtime taking place in the waiting room may have influenced the discontinuation of VR use for the OR transfer and induction.

### Future Research

Future studies aiming to investigate the use or implementation of VR in the perioperative setting should assess the feasibility of the intervention tailored to their organizational context. As mentioned previously, the feasibility of VR during induction of anesthesia could not be well assessed in this study due to the discontinuation rate and feasibility challenges that occurred prior to induction. Future studies should test the effectiveness of various games or software adapted to patient age, interests, and psychological needs. Further practice and research are needed to determine the conditions that would render VR compatible with anesthesia induction. More RCTs would be beneficial to truly assert the efficacy of VR in the perioperative period in comparison to other available technology, including 2D screens and augmented reality.

### Conclusion

In the perioperative setting, from the waiting room until induction, VR may be a valuable tool for temporary distraction to help cope with this setting. While VR demonstrated clinical utility and tolerability, our study found, in the current state of VR implementation at our institution, important feasibility barriers in the waiting room and especially during the induction of anesthesia. Several considerations must be made to address the peculiarities of induction. This study contributes to the growing body of literature about VR in the perioperative process, elucidating important clinical challenges.

## Abbreviations

AR: augmented reality
CSSQ: Child Simulator Sickness Questionnaire
FAS: Faces Anxiety Scale
FPS-R: Faces Pain Scale–Revised
GRS: Graphic Rating Scale
ICC: Induction Compliance Checklist
IV: intravenous
OR: operating room
PACU: postanesthesia care unit
PAED: Pediatric Anesthesia Emergence Delirium
RCT: randomized controlled trial
VR: virtual reality
VR-CORE: VR Clinical Outcomes Research Experts
